# Case Report: Site-specific therapy guided by the 90-gene expression assay in cancer of unknown primary: lenvatinib plus camrelizumab for occult renal cell carcinoma

**DOI:** 10.3389/fonc.2026.1883469

**Published:** 2026-09-24

**Authors:** Liangshan Da, Tai Ma, Jie Da, Wanren Peng

**Affiliations:** Department of Oncology, The First Affiliated Hospital of Anhui Medical University, Hefei, China

**Keywords:** 90-gene expression assay, Cancer of unknown primary, occult renal cell carcinoma, Site-specific therapy, tissue of origin

## Abstract

Cancer of unknown primary (CUP) is primarily treated with empirical chemotherapy, but clinical outcomes remain unsatisfactory. We present the case of a 62-year-old male with asymptomatic left supraclavicular lymphadenopathy. Fine-needle aspiration revealed metastatic poorly differentiated carcinoma. Comprehensive evaluation, including contrast-enhanced computed tomography (CT), fluorodeoxyglucose positron emission tomography/CT (FDG-PET/CT), nasopharyngoscopy, and gastroscopy, failed to identify a definite primary tumor, leading to a diagnosis of CUP. Initial empirical chemotherapy with liposomal paclitaxel and nedaplatin showed limited efficacy and was complicated by grade 3 treatment-related adverse events (TRAEs), resulting in discontinuation due to poor tolerability. Subsequently, a core needle biopsy was performed. Immunohistochemistry suggested metastatic renal cell carcinoma (RCC). A 90-gene expression assay identified the kidney as the most likely primary site, with a similarity score of 82.6. The regimen was then switched to site-specific therapy with lenvatinib plus camrelizumab. The patient tolerated treatment well, experiencing only grade 1 TRAEs. Follow-up contrast-enhanced CT scans demonstrated significant lesion regression (33.1% and 69.6% reductions at 2 and 4 months, respectively), achieving a best response of partial response (PR). In conclusion, the combination of lenvatinib and camrelizumab demonstrated a favorable short-term response and acceptable tolerability in this patient, and renal-like CUP represents a distinct favorable CUP subset.

## Introduction

Cancer of unknown primary (CUP) represents a heterogeneous group of histopathologically confirmed metastatic malignancies. Despite comprehensive clinical, imaging, endoscopic, and laboratory evaluations, the primary tumor site remains unidentified (1). This disease is highly aggressive, with early systemic dissemination and unpredictable metastatic patterns (2). Although CUP accounts for only 1%-3% of all malignancies, it is the fourth leading cause of cancer-related mortality worldwide, with a median overall survival (OS) of 3 to 16 months (3, 4). A key challenge in the management of CUP lies in determining the tissue of origin (ToO), which is essential for identifying favorable subsets that may benefit from site-specific therapy.

Immunohistochemistry (IHC) remains the conventional first-line method for ToO prediction. However, its diagnostic accuracy is approximately 77% (5), and it is particularly unreliable for poorly differentiated or undifferentiated carcinomas. Over the past decade, molecular tumor profiling (MTP) has provided complementary diagnostic approaches, including gene expression profiling (GEP) (6), DNA methylation analysis (7), next-generation sequencing (NGS) (8), and artificial intelligence (AI)-based histopathological assessment (9). Among these, GEP has demonstrated high accuracy in predicting the ToO across multiple cancer types. The 90-gene expression assay, which utilizes real-time quantitative polymerase chain reaction and an AI-driven analytical platform, has shown an overall accuracy of 94.4% for ToO identification in large-scale multicenter studies (10).

For decades, CUP management has primarily relied on empirical platinum-taxane chemotherapy, which offers limited survival benefits with a median OS of less than 12 months (11). In the era of precision oncology, the transition from empirical chemotherapy to site-specific therapy represents a critical paradigm shift, particularly for favorable CUP subsets such as renal-like CUP, which should be treated according to their putative primary tumor sites (12). Previous case series have shown that renal-like CUP, identified through MTP, exhibited favorable responses to renal cell carcinoma (RCC)-specific treatments, such as vascular endothelial growth factor-targeted agents (13, 14). More recently, the phase III Fudan CUP-001 trial demonstrated that site-specific therapy guided by a 90-gene expression assay improved median progression-free survival (PFS) compared with empirical chemotherapy, with a trend toward prolonged OS (15). These findings support the integration of ToO assessment into therapeutic decision-making, although the benefit likely stems from the combination of accurate ToO identification and the application of effective modern site-specific therapies.

Herein, we report a patient with CUP who presented with lymph node metastasis, in whom a 90-gene expression assay identified a renal origin. After disease progression and severe treatment-related adverse events (TRAEs) during empirical chemotherapy, the patient achieved significant tumor regression with lenvatinib plus camrelizumab according to the putative primary site.

## Case description

In early November 2025, a 62-year-old man was referred to our institution for asymptomatic left supraclavicular lymphadenopathy. Fine-needle aspiration (FNA) at an outside hospital had revealed metastatic poorly differentiated carcinoma. Laboratory tests showed a mild elevation in serum cytokeratin fragment antigen 21-1 (CYFRA21-1), whereas other common serum tumor markers remained within normal reference ranges. Epstein-Barr virus (EBV) testing was negative. Contrast-enhanced computed tomography (CT) (November 17, 2025) revealed multiple enlarged lymph nodes in the left supraclavicular, mediastinal, and retroperitoneal regions (**Figure 1A**). Fluorodeoxyglucose positron emission tomography/CT (FDG-PET/CT) demonstrated metabolically active lymph nodes (maximum standardized uptake value [SUVmax] = 5.22) in these areas, with no other FDG-avid lesions detected (**Figure 1E**). Gastroscopy and nasopharyngoscopy showed no evidence of a primary tumor. A clinical diagnosis of CUP was established. Limited tissue obtained via FNA precluded additional immunohistochemical stains and molecular testing, which were essential for identifying the ToO and guiding precision therapy. We strongly recommended a core needle biopsy. Despite counseling the patient on the potential benefits of core needle biopsy, as well as the expected risks and limitations of empirical treatment, the patient developed marked anxiety after the diagnosis and requested immediate treatment. Palliative systemic therapy was initiated on November 21, 2025, with intravenous liposomal paclitaxel (210 mg) and nedaplatin (110 mg) administered every 21 days. The patient developed grade 3 TRAEs, including arthralgia, myalgia, fatigue, and anorexia, leading to treatment discontinuation due to poor tolerability. Follow-up CT on December 16, 2025 (**Figure 1B**) showed a slight increase in lesion size from the prior scan.

**Figure 1:**
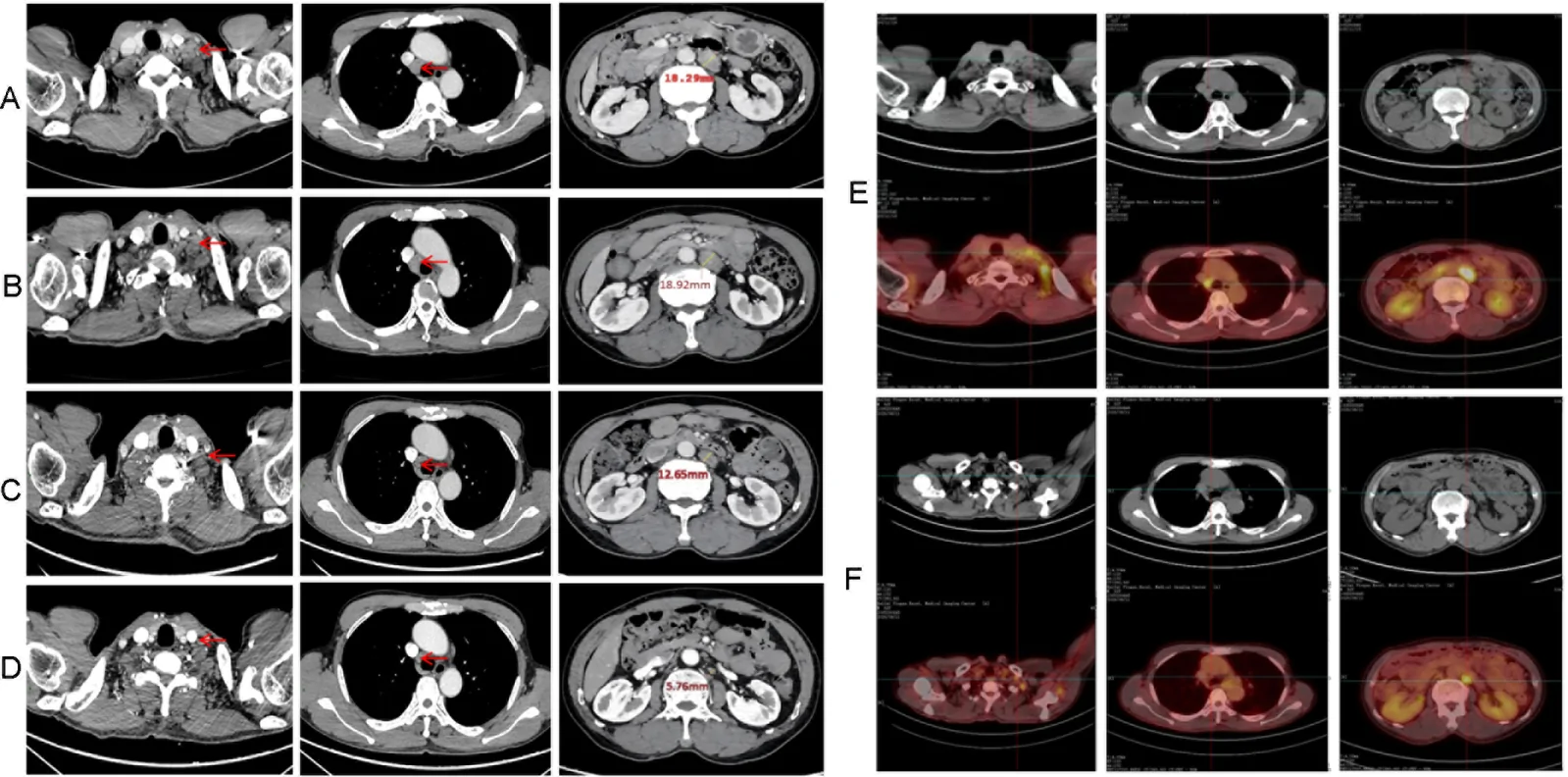
Computed tomography (CT) and fluorodeoxyglucose positron emission tomography/CT (FDG-PET/CT) images before and after treatment. **(A)** Baseline CT; **(B)** CT after one cycle of empirical chemotherapy; **(C)** CT after two cycles of site-specific therapy; **(D)** CT after four cycles of site-specific therapy; **(E)** Baseline FDG-PET/CT; **(F)** FDG-PET/CT after eight cycles of site-specific therapy.

An ultrasound-guided core needle biopsy was subsequently performed. Hematoxylin and eosin (H&E) staining revealed metastatic poorly differentiated carcinoma (**Figure 2A**). Immunohistochemical analysis was positive for PAX-8 (**Figure 2B**), PAX-2, CAIX (**Figure 2C**), CD10 (**Figure 2D**), vimentin, CK7, SATB2, and villin; negative for p40, CK5/6, p63, CK20, CEA, CDX-2, and TTF-1. This immunoprofile suggested metastatic renal cell carcinoma (mRCC), raising a strong suspicion of occult RCC. A 90-gene expression assay was further performed to confirm the ToO. The assay demonstrated that RCC yielded the highest similarity score of 82.6, markedly higher than those of neuroendocrine tumors (5.1) and mesothelioma (2.2). According to the interpretation criteria of this assay, a similarity score ≥45.3 indicates that the submitted tumor sample most likely originates from the corresponding tissue type. The score in this case was substantially above the cutoff value, strongly supporting a renal primary (**Figure 3**).

**Figure 2:**
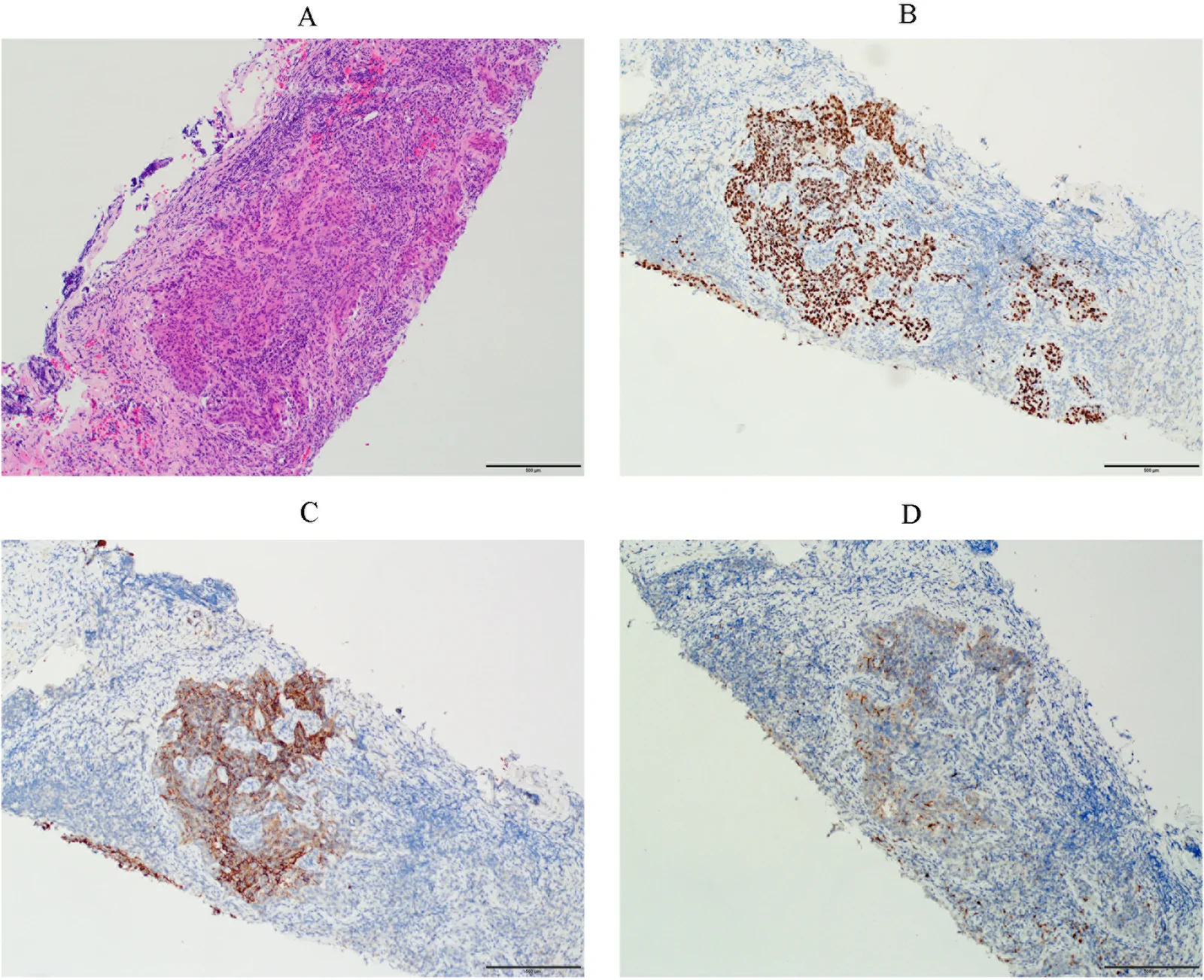
Histopathological and immunohistochemical findings of the left supraclavicular lymph node. **(A)** Hematoxylin and eosin (H&E) staining. **(B)** PAX-8 positive; **(C)** CAIX positive; **(D)** CD10 positive. Original magnification: ×100.

**Figure 3:**
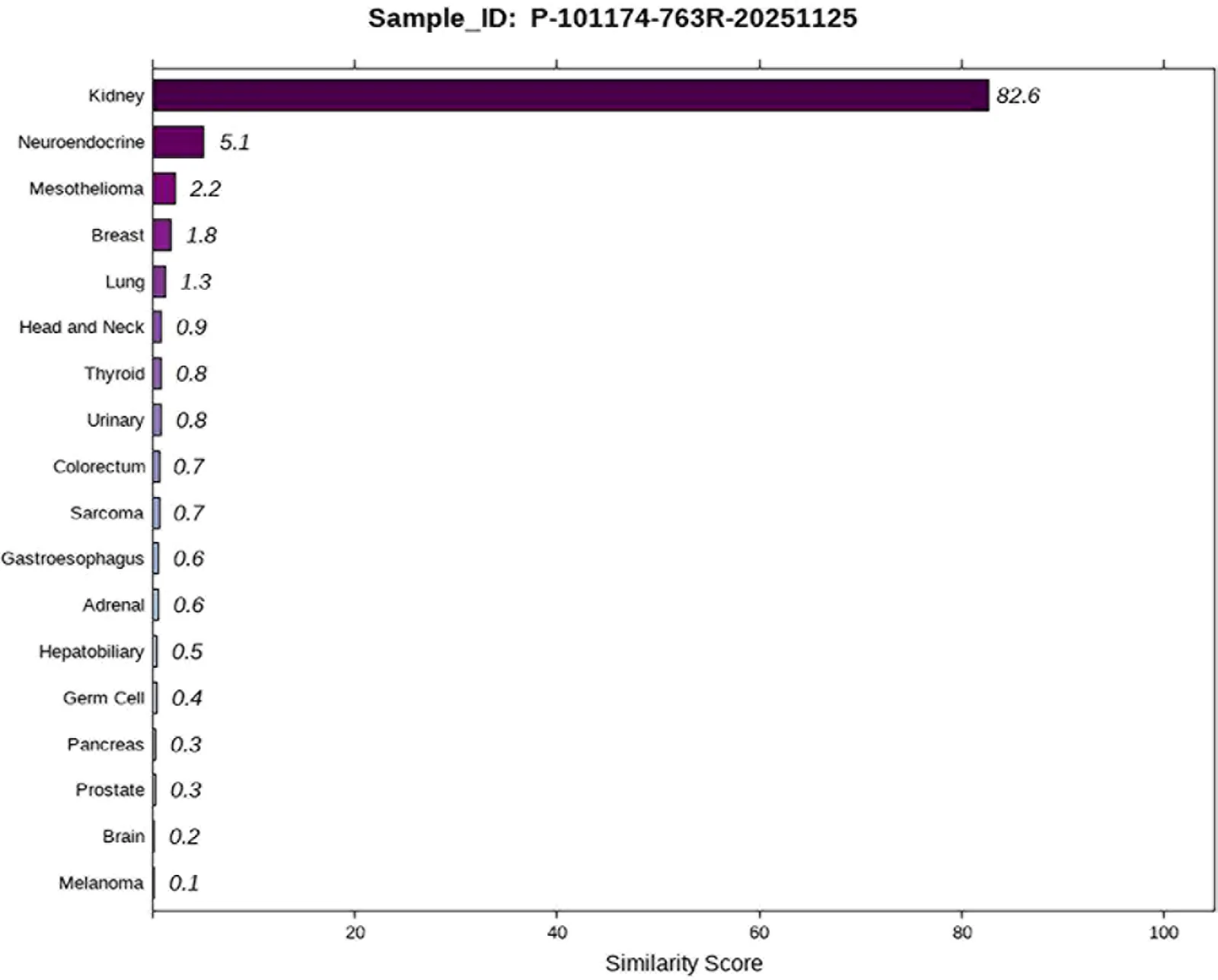
Tissue of origin (ToO) confirmed by 90-gene assay. The 90-gene expression assay of the left supraclavicular lesion identified the kidney as the ToO, with the highest similarity score of 82.6.

Treatment was then switched to site-specific combination therapy with lenvatinib (12 mg orally once daily) and camrelizumab (200 mg intravenously on day 1, every 21 days). Follow-up CT on February 7, 2026 (**Figure 1C**) and April 8, 2026 (**Figure 1D**) showed tumor reductions of 33.1% and 69.6% in target lesions, respectively, compared with the post-chemotherapy baseline. On August 11, 2026, FDG-PET/CT demonstrated marked shrinkage and even resolution of the aforementioned lymph node lesions, with a substantial reduction in FDG activity of the mediastinal lymph nodes (**Figure 1F**). According to Response Evaluation Criteria in Solid Tumors (RECIST) version 1.1, the best response achieved was partial response (PR). The regimen was well tolerated, with only grade 1 TRAEs observed, including reactive cutaneous capillary endothelial proliferation, elevated alanine aminotransferase and aspartate aminotransferase, and leukopenia. The patient remains stable and asymptomatic, with ongoing regular follow-up and treatment. The main clinical timeline is summarized in **Figure 4**.

**Figure 4:**
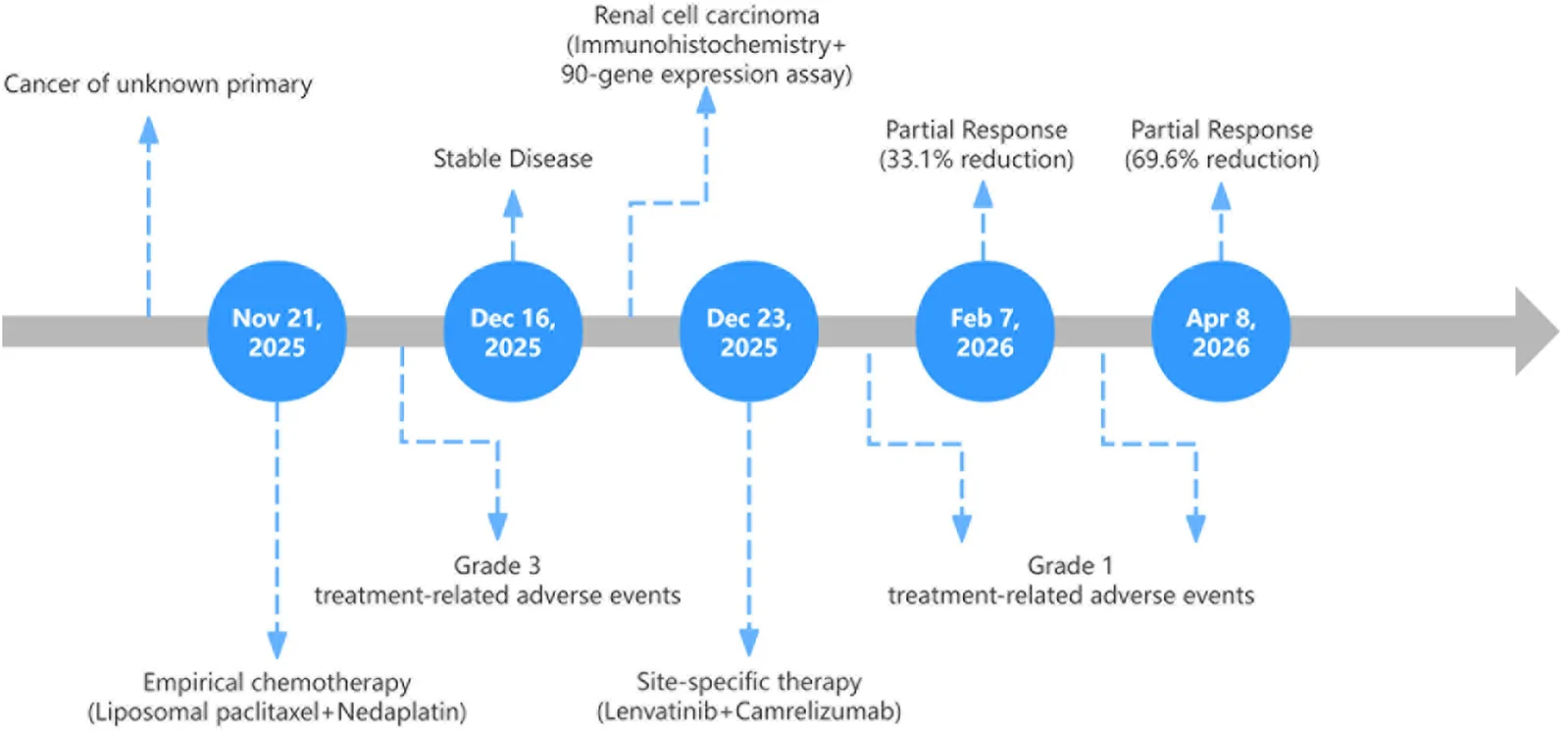
Patient diagnostic and therapeutic timeline.

## Discussion

CUP encompasses a heterogeneous group of metastatic malignancies, and occult RCC represents a small but clinically relevant subgroup within this disease spectrum. Greco et al. reported that a renal primary could be identified or predicted in approximately 4.4% of CUP patients in their cohort (13). The 2023 European Society for Medical Oncology (ESMO) guidelines explicitly classify carcinomas with renal-like histological and immunohistochemical profiles as favorable CUP subtypes, warranting site-specific therapy analogous to mRCC (12). mRCC-specific treatments, including tyrosine kinase inhibitors (TKIs) and immune checkpoint inhibitors (ICIs), differ fundamentally from empirical chemotherapy in both mechanisms and efficacy, and generally yield superior outcomes. Since no agents overlap between these two regimens, identifying this subset is critical for treatment decision-making.

The 90-gene expression assay played a critical complementary role in this case. While IHC provided initial evidence of renal origin, the assay delivered quantitative molecular confirmation with a high similarity score (82.6) for RCC, substantially exceeding the scores for alternative diagnoses. This is particularly relevant in cases of occult RCC, where the primary tumor may be radiologically undetectable due to spontaneous regression or early systemic dissemination followed by microenvironmental suppression of primary tumor growth (1, 16–18). This assay provided supplemental molecular evidence and reduced diagnostic uncertainty in this case where IHC suggested RCC with no radiological evidence of a primary tumor, informing therapeutic decision-making.

According to the International Metastatic Renal Cell Carcinoma Database Consortium (IMDC) prognostic classification system, this patient was classified into the intermediate-risk group. The selection of lenvatinib plus camrelizumab was based on comprehensive considerations including drug availability, affordability, and available clinical evidence. Although pembrolizumab combined with lenvatinib represents the globally recognized first-line standard regimen for mRCC supported by the phase III CLEAR trial (19), pembrolizumab has not been approved for mRCC in China, with no medical insurance coverage or patient assistance programs. The full out-of-pocket cost was unaffordable for this patient. In contrast, camrelizumab is a widely used programmed death 1 (PD-1) inhibitor in China with favorable efficacy and safety profiles and a relatively lower price. Despite the absence of large randomized controlled trials supporting lenvatinib combined with camrelizumab, a multicenter phase II basket trial reported that camrelizumab combined with famitinib achieved an objective response rate (ORR) of 60.5% and a median PFS of 14.6 months in the overall mRCC cohort. Superior outcomes were observed in treatment-naive patients, with an ORR of 84.6% and an unreached median PFS (20). These data, combined with the established efficacy of TKI-ICI combinations in mRCC, provided potential clinical evidence for the off-label use of lenvatinib plus camrelizumab in this patient with renal-like CUP.

To our knowledge, this is the first report of camrelizumab plus lenvatinib for occult RCC. Nevertheless, several limitations of this case cannot be ignored. First, although molecular evidence strongly supported renal origin, we failed to establish definite pathological subtyping. Second, programmed death-ligand 1 (PD-L1) testing and comprehensive NGS were not performed, which limited further molecular characterization and individualized drug selection. Third, the follow-up time after site-specific therapy is only around eight months, and long-term survival endpoints including PFS and OS remain immature. Lastly, as a single case report, these findings cannot be generalized beyond the renal-like CUP subset.

In conclusion, the combination of lenvatinib and camrelizumab demonstrated a favorable short-term response and acceptable tolerability in this patient. This case highlights the complementary diagnostic value of the 90-gene expression assay in confirming renal origin when IHC is suggestive but imaging is inconclusive. Furthermore, it supports renal-like CUP as a distinct favorable subset within the CUP spectrum.

## Patient perspective

After administration of lenvatinib plus camrelizumab, the patient reported significant shrinkage of the cervical mass and tolerated the treatment well. He felt much better overall and had a noticeably improved quality of life. The patient was satisfied with the treatment efficacy and consistently followed up as arranged.

## Data Availability

The original contributions presented in the study are included in the article/supplementary material. Further inquiries can be directed to the corresponding author.
